# Mantle Cell Lymphoma Mimicking Parotid Neoplasm: A Rare Case Report

**DOI:** 10.22038/ijorl.2025.88661.3973

**Published:** 2026

**Authors:** Tejaswi Gupta, Sanjeev Yadav, Ahmed Aseem Naseem, Sanjeev Kumar Singh, Rashmi Rashmi

**Affiliations:** 1 *Department of Otorhinolaryngology (ENT), UPUMS, Saifai, Etawah, 206130. India.*; 2 *Department of Otorhinolaryngology (ENT), Dr KNS Memorial Institute of Medical Science. Barabanki, Pin code: 225001, India. *; 3 *Department of Pathology, UPUMS, Saifai, Etawah, 206130. India.*

**Keywords:** Mantle Cell Lymphoma, Parotid gland, Immunohistochemistry (IHC)

## Abstract

**Introduction:**

Mantle cell lymphoma (MCL) is a rare and aggressive B-cell non-Hodgkin lymphoma, commonly affecting lymph nodes, spleen, bone marrow, and gastrointestinal tract. Salivary gland involvement, especially in the parotid gland, is unusual and often mimics benign conditions, complicating diagnosis.

**Case Report:**

A 58-year-old male presented with a painless, progressively enlarging swelling in the right preauricular region without facial nerve involvement. Imaging revealed a mass within the parotid gland, leading to superficial parotidectomy. Histopathology confirmed mantle cell lymphoma. Immunohistochemical studies showed positivity for CD20, BCL2, CD5, and Cyclin D1; negativity for CD23, CD10, BCL6, and MUM1; and scattered CD3-positive T lymphocytes. The Ki-67 proliferation index was approximately 40%, indicating intermediate proliferative activity. Whole-body PET-CT revealed additional metabolically active lesions suggestive of systemic disease. The patient was started on bendamustine and rituximab chemotherapy.

**Conclusion:**

This case highlights that parotid swellings may conceal systemic lymphomas, and misleading cytology can delay diagnosis. Clinicians should consider MCL in atypical parotid lesions to ensure early systemic therapy.

## Introduction

Mantle cell lymphoma (MCL) is a rare and aggressive form of B-cell non-Hodgkin lymphoma, accounting for approximately 6–8% of adult cases (1). It is defined by the chromosomal translocation t (11;14) (q13; q32), which leads to overexpression of cyclin D1, contributing to uncontrolled cellular proliferation (2). MCL involving salivary glands, particularly the parotid gland, is exceedingly uncommon and can mimic both benign and malignant salivary gland tumors, posing diagnostic challenges (3). Most patients present with a painless swelling in the parotid region. Since Fine-needle aspiration cytology (FNAC) often yields inconclusive results, histopathology and immunohistochemistry remain essential for definitive diagnosis (4). Early recognition is important, as MCL requires distinct therapeutic approaches and has a different prognosis compared to other parotid neoplasms.

## Case Report

A 58-year-old man presented to the outpatient department with a gradually enlarging swelling in the right preauricular region for the past 4–5 months. The swelling was painless and not associated with facial weakness or neurological symptoms. There was no history of fever, night sweats, or weight loss suggestive of B-symptoms.

 On physical examination, no cervical lymphadenopathy was noted, but bilateral inguinal lymph node enlargement was observed.

**Fig 1:**
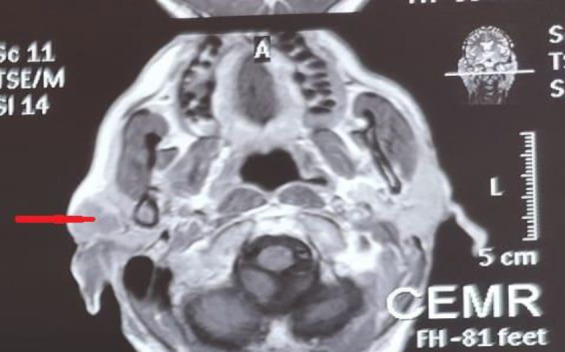
Contrast MRI neck: Axial post contrast T1 Weighted sequence of the neck showing Right side of parotid mass (Red arrow). The lesion appears **mildly hypointense on T1-weighted images** with mild peripheral enhancement.

Fine-needle aspiration cytology (FNAC) from both the inguinal nodes and the right parotid swelling revealed reactive hyperplasia, illustrating the limitation of cytology in diagnosing mantle cell lymphoma (MCL) and representing a key diagnostic pitfall. Hemoglobin, total leukocyte count, and platelet counts were within normal limits, and peripheral smear showed normocytic, normochromic red blood cells without atypical lymphoid cells. Serum lactate dehydrogenase (LDH) levels were mildly elevated at 270 U/L.Contrast-enhanced magnetic resonance imaging (MRI) of the neck revealed a soft tissue mass in the right parotid gland, demonstrating intermediate to mildly hypointense signals on T1- and T2-weighted sequences with mild peripheral enhancement after contrast administration (Fig. 1).

Considering the persistent parotid lesion, the patient underwent superficial parotidectomy under general anesthesia. A modified Blair incision was used, and the superficial musculoaponeurotic system (SMAS) flap was elevated (Fig. 2a). The facial nerve and its branches were identified and preserved (Fig. 2b). The excised superficial lobe was sent for histopathological examination.

**Fig 2. A:**
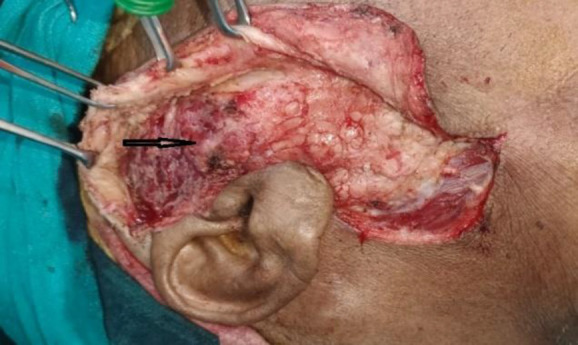
Intraoperative Picture.: After elevating SMAS flap (Superficial Musculoaponeurotic system flap), right side parotid mass (Black arrow)

**Fig 2. B:**
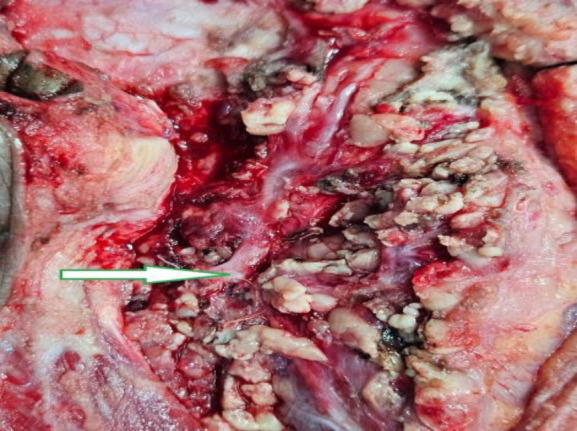


Histopathological examination of the right parotid specimen confirmed mantle cell lymphoma. Microscopy revealed infiltration of salivary gland parenchyma by small- to medium-sized monomorphic atypical lymphoid cells with a high nuclear-to-cytoplasmic ratio, hyperchromatic irregular nuclei, clumped chromatin, inconspicuous nucleoli, and scant cytoplasm. Tumor cells infiltrated surrounding adipose tissue and skeletal muscle (Fig. 3a, 3b).

Immunohistochemistry (Table 1, Fig. 4) showed positivity for CD20, CD5, BCL2, and Cyclin D1, while CD23, CD10, BCL6, and MUM1 were negative. Scattered CD3-positive T lymphocytes were noted. The Ki-67 index was approximately 40%, indicating intermediate proliferative activity.

**Fig 3. A:**
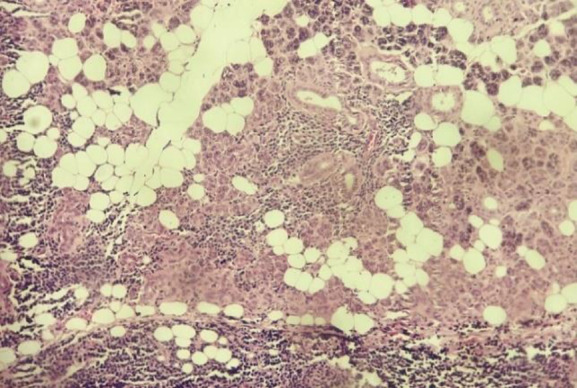
100X, (Hematoxylin and Eosin staining) salivary gland tissue is infiltrated by atypical lymphoid cells

**Fig 3. B:**
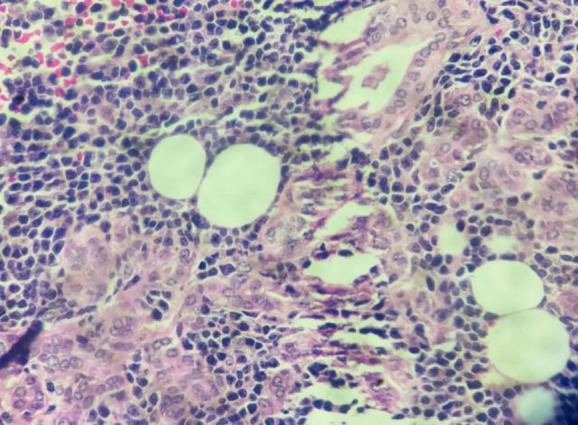
400 X (Hematoxylin and Eosin staining) monomorphic atypical lymphocyte with high nucleocytoplasmic ratio and hyperchromatic nuclei infiltrating into surrounding tissue.

**Table 1:** Immunohistochemical markers

**Markers**	**Interpretations**
CD20	Positive
BCL2	Positive
CD5	Positive
Cyclin D1	Positive
CD3	Positive in scattered T lymphocytes
CD23	Negative
CD10	Negative
BCL6	Negative
MUM1	Negative
Ki67	40%

**Fig 4. A:**
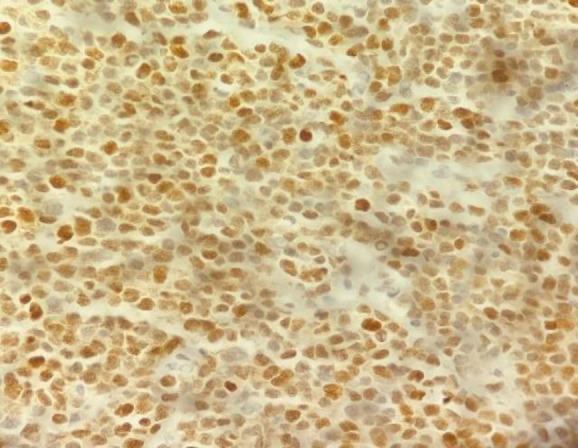
Immunohistochemical markers. Cyclin D1 immunoreactivity is positive

**Fig 4 B:**
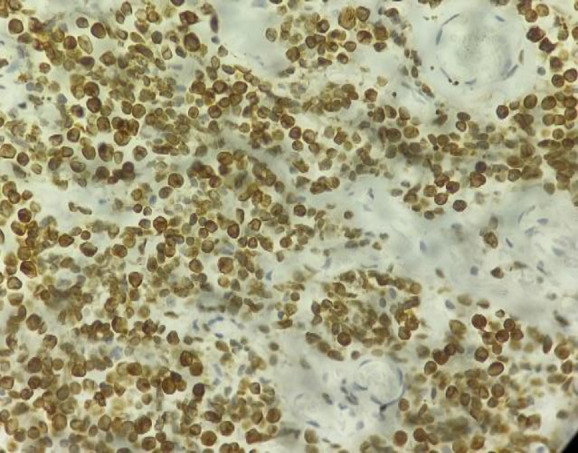
BCL2 positive

**Fig 4. C:**
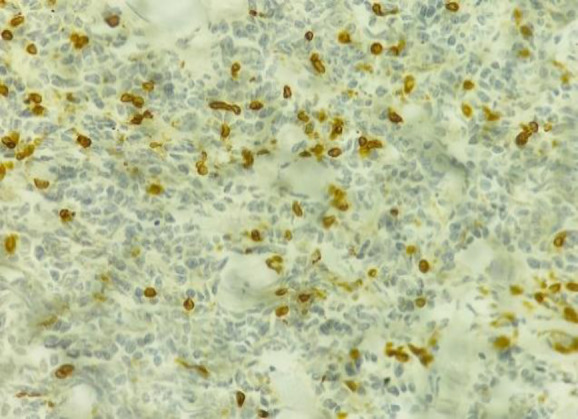
CD3 positive in scattered T cells

**Fig 4. D:**
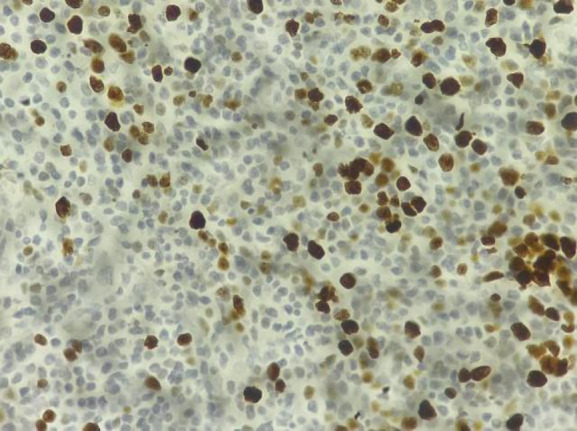
Ki67 40 percent

Whole-body positron emission tomography (PET-CT) demonstrated increased FDG uptake in the bilateral palatine tonsils and parotid regions, along with metabolically active lesions in the ascending colon, duodenum, and ileum. Interestingly, the previously enlarged inguinal nodes showed no abnormal uptake, supporting their reactive rather than malignant nature.

The patient was referred to oncology and initiated on combination chemotherapy with bendamustine and rituximab (BR regimen). At 15 months of follow-up, he remained clinically disease-free; however, given the aggressive behavior of MCL, close long-term surveillance was advised.

## Discussion

Mantle cell lymphoma (MCL) is a rare, aggressive B-cell non-Hodgkin lymphoma arising from the mantle zone of lymphoid follicles. It predominantly affects older males and is often diagnosed at an advanced stage, involving lymph nodes, bone marrow, spleen, and the gastrointestinal tract (5,6). 

Parotid gland involvement, as in this case, is exceedingly uncommon, emphasizing its rarity and the diagnostic challenges it poses in the head and neck region (7,8). Prior reports consistently describe MCL presenting as painless swelling in older males, aligning with our patient’s demographics (8).

Diagnosis relies on histopathology and immunophenotypic profiling. Tumor cells typically express CD20, CD5, and Cyclin D1, while lacking CD10 and Bcl-6. Cyclin D1 is central to MCL diagnosis, as its overexpression drives cell cycle dysregulation. 

In addition, SOX11 expression serves as a critical adjunct marker, especially in Cyclin D1-negative or atypical cases, confirming mantle zone derivation and supporting definitive diagnosis. Diffuse Cyclin D1 positivity, together with SOX11, thus provides strong diagnostic certainty (9,10).

The Ki-67 proliferation index is a key prognostic marker, with values above 30% indicating aggressive disease. These parameters, along with age, performance status, lactate dehydrogenase (LDH) level, and leukocyte count, form the Mantle Cell Lymphoma International Prognostic Index (MIPI), which stratifies patients into risk groups. Extranodal involvement, as seen here, further impacts prognosis (9,10).

This case is particularly noteworthy because the initial cytology was misleading, contralateral involvement developed rapidly postoperatively, and systemic spread was detected only on PET-CT. These findings highlight diagnostic pitfalls when MCL presents as a parotid swelling and underscore the importance of comprehensive systemic evaluation, including advanced imaging, for accurate staging and management.

Treatment strategies have evolved substantially. Chemoimmunotherapy remains the mainstay, with bendamustine-rituximab (BR) preferred in older patients due to favorable progression-free survival and tolerability (5). 

In relapsed or refractory disease, targeted therapies such as Bruton's tyrosine kinase (BTK) inhibitors—including ibrutinib, acalabrutinib, and zanubrutinib—have shown promising efficacy. Chimeric antigen receptor T-cell (CAR-T) therapy and maintenance rituximab are additional approaches that improve long-term outcomes (10). These advances highlight the importance of a personalized, risk-adapted approach based on prognostic indices, patient characteristics, and disease biology.

## Conclusion

This case highlights the diagnostic challenges of mantle cell lymphoma presenting in an atypical site such as the parotid gland, where initial cytology may be misleading and contralateral or systemic involvement can occur rapidly. It underscores the importance of comprehensive systemic evaluation, including advanced imaging, for accurate staging and management. Cyclin D1 and SOX11 remain cornerstone markers for definitive diagnosis, while the Ki-67 index and MIPI score provide essential prognostic information. Awareness of these diagnostic and prognostic tools, combined with knowledge of emerging therapies—including BTK inhibitors, CAR-T therapy, and maintenance rituximab—can guide timely, personalized management and improve patient outcomes
